# Insights into rhodopsin molecular evolution from mice with “humanized” Phe-88 to Leu substitution

**DOI:** 10.21203/rs.3.rs-7606538/v1

**Published:** 2025-10-14

**Authors:** Feifei Wang, Alexander V. Kolesnikov, Shinya Sato, Aneal Singh, Clint L. Makino, Pere Garriga, Vladimir J. Kefalov

**Affiliations:** 1 Grup de Biotecnologia Molecular i Industrial, Centre de Biotecnologia Molecular, Departament d’Enginyeria Quimica, Universitat Politècnica de Catalunya-Barcelona Tech, Edifici Gaia, Rambla de Sant Nebridi 22, 08222 Terrassa, Catalonia, Spain; 2 Gavin Herbert Eye Institute – Center for Translational Vision Research, Department of Ophthalmology and Visual Sciences, University of California Irvine, Irvine, California, 92697, USA; 3 Department of Physiology and Biophysics, University of California Irvine, Irvine, California, 92697, USA; Department of Pharmacology, Physiology and Biophysics, Boston University Chobanian and Avedisian School of Medicine, Boston, Massachusetts, 02118, USA

**Keywords:** Rhodopsin, Phototransduction, Molecular evolution, Nocturnal vision, Diurnal vision

## Abstract

The function of rod photoreceptors as dim light photon detectors depends critically on the molecular properties of their visual pigment, rhodopsin. The structure of rhodopsin has evolved under selective pressure to light conditions of different spectral composition and overall intensity. One notable example is the switch of mammalian species from nocturnal to diurnal environments. Comparison of the rhodopsins of the nocturnal mouse and the diurnal human reveals high sequence similarity, with only 18 distinct amino acids. Here, we examined the role of one of these, mouse phenylalanine (F) vs. human leucine (L) at position 88, in modulating the molecular properties of rhodopsin and the function of rods by generating F88L rhodopsin knock-in mouse. Our detailed *in vitro* analysis of the physicochemical properties of this mutant F88L rhodopsin showed a higher conformational stability and more efficient chromophore regeneration compared to the WT mouse pigment. We also found that the decay of metarhodopsin II in the F88L mutant occurred significantly faster than in the WT. However, despite these molecular changes, the visual function of knock-in mutant mice carrying the F88L mutation was not significantly altered by this amino acid change. These findings demonstrate the role of the F88L evolutionary switch in enhancing the stability and regeneration of rhodopsin towards visual function in diurnal human rods over nocturnal mouse rods. Our results provide new insights into the molecular evolution of rhodopsin in vertebrates.

## Introduction

Genetic evolution is a core mechanism of life, driving biodiversity and enhancing the adaptation of organisms to their environments^1^. The diversity of species is driven by genetic changes such as mutations, recombinations, and other variations brought about by mechanisms of genetic evolution^2,3^. The continuous evolution of genes serves to adapt to different living environments. For example, eagles have a visual range of up to 3 kilometers^4^ and owls have dim light vision far better than that of humans^5^. This adaptability optimizes visual function and improves the survival chances of species. Genetic evolution has also driven the improvement of protein functions. For example, early mammals had dichromatic vision, which included only blue opsins responsible for short wavelengths and red opsins responsible for long wavelengths^6,7^. Through genetic evolution, primates developed trichromatic vision by adding green opsins responsible for medium wavelengths, thereby achieving a richer visual perception^8,9^. In addition to elucidating the patterns of cellular evolution, studies of visual function in diverse species could help reveal the mechanisms of hereditary diseases and provide new approaches for gene-level treatments.

As the visual systems of vertebrates evolved, rhodopsin proteins continuously adapted and adjusted to cope with different environments. Rhodopsin, a member of the G protein-coupled receptor family, primarily functions to provide vision in dim light environments, allowing vertebrates to survive in low-light conditions such as at night or in the deep-sea^10^. Adaptation and evolution have resulted in a great diversity of rhodopsins. For example, the silver spinyfin, which lives in the deep sea, possesses 38 specialized rod opsins, possibly enabling it to detect bioluminescence in deep waters^11^. As another example, the nocturnal mouse retina is strongly rod-dominated, with rods outnumbering cones by roughly 35:1^12^. By analyzing and comparing genetic differences, we can better understand the mechanisms of evolution and provide new insights into the molecular mechanisms that modulate photoreceptor function and vision overall.

**Fig. 1.**
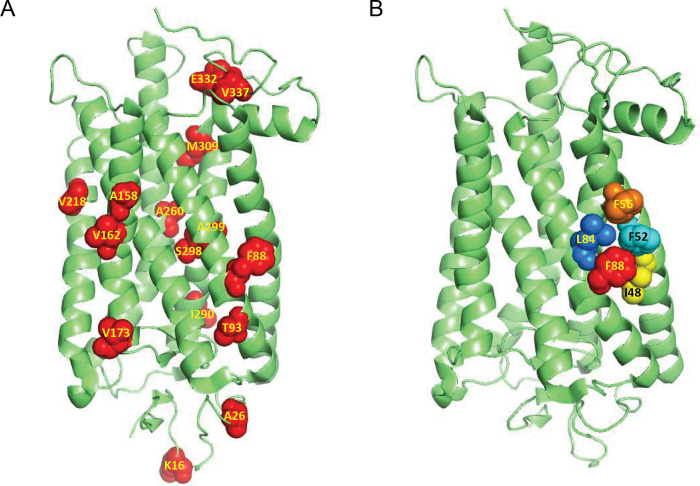
Localization of the F88 site in rhodopsin. (**A**) Three-dimensional structure of bovine rhodopsin^63^ showing most of the positions that are different between mouse and human rod pigments. (**B**) Three-dimensional structure of bovine rhodopsin (same as in **A**) indicating the F88 amino acid side chain and a cluster of aromatic amino acids (mainly Phe) in its vicinity.

Most biochemical studies on rod visual pigments use either mouse or bovine rhodopsin as models^13,14^. The rhodopsin amino acid sequences of cows and mice are highly similar to that of humans. However, there are a total of 18, mostly hydrophobic, amino acid residues different between mouse and human rod pigments that are distributed throughout their entire sequences (Fig. 1A). It is not clear whether one or more of these distinct sites contribute to the structural and functional differences between the rhodopsins of nocturnal (mouse) and diurnal (human) species. One of these amino acid differences is at position 88 on the surface of the 2^nd^ transmembrane domain of rhodopsin: mice have phenylalanine (F), while humans have leucine (L) at the equivalent site. The position is surrounded by several hydrophobic residues not different between the two pigments (Fig. 1B). Here, we aimed to understand how this amino acid affects the molecular properties of rhodopsin and whether it influences the function of mouse *vs.* human rod photoreceptors. We addressed these questions using mice with an F88L rhodopsin substitution and investigated the potential effects of this point mutation on rhodopsin biochemical properties and rod photoreceptor function.

## Methods

### Generation of rhodopsin F88L knock-in mouse line

CRISPR/Cas9-based genome editing was performed as previously described^15^. The guide RNA (gRNA) for Cas9 was designed based on proximity to the rhodopsin target amino acid codon and synthesized from respective mouse DNA sequence (GACCTCTTCATGGTCTTCGG) using the MEGAshortscript T7 Transcription Kit (Thermo Fisher Scientific). The gRNA was tested for cutting efficiency in cell culture. The validated gRNA and Cas9 protein were then microinjected into the pronuclei of C57Bl/6J-0.5-dpc (days post coitum) zygotes along with the donor DNA, a 190-bp single-stranded oligodeoxynucleotide carrying the codon substitution for rhodopsin G90D mutation (which was the original goal of the project). Embryos were then transferred into the oviduct of pseudo-pregnant female. Pups were delivered ~20 days after microinjection. Tissues from 10-day postnatal (P10) pups were collected by toe/tail biopsy for identification of the targeted allele. The genotyping was initially done by standard PCR-amplification of a 323-bp rhodopsin gene fragment using the forward (TTATGTGCCCTTCTCCAACG) and reverse (CAAAGAAGCCCTCGAGATTACA) primers followed by Sanger sequencing. Subsequent genotyping assay was developed and performed by Transnetyx. One of the heterozygous male founders containing a F88L mutation (without G90D mutation) was kept for this study. It was crossed with 129S2/Sv female mice (which carry the Leu-450 variant of RPE65; Charles River Laboratories) and the offspring were then intercrossed to obtain homozygosity of both F88L and Leu-450 *Rpe65* alleles. Control wild type rhodopsin animals on the same Leu-450 *Rpe65* genetic background were also derived from those breedings. All animal experiments were carried out in accordance with the NIH Guide for the Care and Use of Laboratory Animals and the ARVO Statement for the Use of Animals in Ophthalmic and Vision Research and approved by Institutional Animal Care and Use Committees of UC Irvine, Universitat Politècnica de Catalunya, and Boston University. All procedures involving animals were conducted in accordance with ARRIVE guidelines^16^.

### Rhodopsin purification

WT and F88L mutant rhodopsins were purified from frozen, dark-adapted retinas of 3–4-month-old mice under dim red light^17^. Briefly, retinas were placed in 2 mM sodium phosphate (NaPi) buffer containing 1 % (w/v) dodecyl maltoside (DM) at pH 6.0, gently nutating for 1 h at 4 °C, until they were completely dissolved. The sample was subsequently centrifuged at 4,000 g, for 25 min at 4 °C, and the supernatant was retained. The pigments were purified by immunoaffinity chromatography using Rho-1D4 antibody coupled to CNBR-activated Sepharose beads. Afterwards, they were washed three times with NaPi, pH 6.0, containing 0.05% (w/v) DM. Rhodopsin was eluted using the same buffer containing 100 μM 9-mer peptide (TETSQVAPA) corresponding to the last 9 amino acids of the C-terminal domain of rhodopsin. The yield of the purification was assessed by UV-visible spectrophotometry of the purified samples on a Varian Cary 100 UV-Vis spectrophotometer from 250 nm to 650 nm. The purification process was conducted in the dark or under dim red light and at 4 °C during the whole purification process.

### Rhodopsin photobleaching and acidification behavior

The samples were analyzed for their photobleaching and acidification behavior to determine whether the mutation had perturbed the normal first step of the visual phototransduction pathway^18^. Acidification is used to determine potential changes in the stability of the Schiff base linkage after illumination what would suggest the presence of an unstable active Metarhodopsin II (Meta II) conformation of the receptor. To this aim, the UV-Vis spectra of purified rhodopsin were measured in the dark (dark spectrum) first, and then after photobleaching with a Dolan Jenner FIBER-LITE-MI-150 light source, equipped with an optic fiber guide and a 495-nm cut-off filter, for 30 s at 20 °C (light spectrum). Finally, the sample was acidified with 2 M H_2_SO_4_, to a final concentration of 40 mM (acid spectrum). UV-Vis spectra were measured on a Varian Cary 100 UV-Vis spectrophotometer in the 250 nm to 650 nm range, with a scan speed of 400 nm/min.

### Thermal and chemical stabilities of rhodopsin

The thermal stability of the purified WT and F88L mutant rhodopsins at different temperatures was examined in darkness^19^. The assay measured the decay of the visible band at 500 nm over time. In this way, the stability of the chromophore-bound conformation of rhodopsin was determined, rather than the overall thermal stability of the protein that could be detected by other methodologies, such as microcalorimetry. The first spectrum was recorded at three temperatures (48 °C, 55 °C, or 60 °C), and the spectrophotometer was set to measure cycles every 2 min with a scan speed of 400 nm/min to monitor the spectral changes with time at the selected temperature. The results were analyzed by plotting the change in absorbance at 500 nm versus time and fitting the obtained curve with an exponential function. In this way, the half-time (t_1/2_) of the decay process could be derived from the fitted curve.

The chemical stability was measured in the dark by adding hydroxylamine, to a final concentration of 50 mM, to the purified rhodopsin samples. The reactivity towards hydroxylamine in the dark reflects the conformational stability and the accessibility of the retinal binding pocket and the Schiff base in the dark state. Therefore, this assay is used to determine the degree of structural compactness around the retinal binding domain that is high in the correctly folded WT (reflected in small changes in absorbance in the dark with time) but that is sometimes altered as a result of mutation reflecting a less compact structure. Spectra were recorded over time with a 2-min interval to monitor the spectral changes, and the spectral data were analyzed by plotting the absorbance decrease at 500 nm that would reflect the amount of rhodopsin left in the sample at the end of the experiment.

### Rhodopsin regeneration

The regeneration ability of the photobleached rhodopsin samples was measured by UV-Vis spectrophotometry, essentially as previously described^18^. First, the spectrum of rhodopsin in the dark was recorded. Then, exogenous 11-*cis-*retinal, from a concentrated stock in ethanolic solution, was added to the sample (to a 1:2 rhodopsin/retinal molar ratio) and the corresponding spectrum was measured. In the next step, the sample was photobleached for 30 s as previously described (see Photobleaching section), and the corresponding photobleached spectrum was recorded immediately afterwards. The pigment regeneration process was followed by collecting a total of 50 spectral scans with a time lapse of 2 min between them and a scan speed of 400 nm/min. The absorbance change at 500 nm was plotted as a function of time and the experimental data points were fitted to an exponential curve, from which the t_1/2_ of the process was derived.

### Retinal release from rhodopsin

Fluorescence spectroscopy was used to follow the retinal release process from the photobleached rhodopsin. Under our experimental conditions, this process closely matched the active Meta II decay. The rates of retinal release from the photobleaching rhodopsin were measured by recording the fluorescence changes on a Photon Technologies International Quanta Master 4 Spectrofluorometer according to a previously described method^20^. Briefly, the fluorescence data were obtained with the following parameters: excitation wavelength of 295 nm, emission wavelength of 330 nm, and the sample was measured for 2 s. A beam shutter was used to pause data acquisition for 28 s between data measurements to avoid photobleaching of the protein. When the fluorescence reading in the dark stabilized, the sample was illuminated for 30 s in the spectrofluorometric cuvette using the same light source indicated above, and the fluorescence increase with time was continuously monitored. The fluorescence data were fitted to an exponential function and the t_1/2_ of the process was determined.

### Meta III formation and decay

Upon photoactivation, rhodopsin undergoes a conformational change to form the active Meta II intermediate as a result of the 11-*cis* to all-t*rans* isomerization of the retinal chromophore. Following this conformational change, the Schiff base linkage between all-*trans* retinal and opsin becomes reprotonated, leading to the formation of Metarhodopsin III (Meta III) with a maximum absorption band at 465 nm. This longer-living photoproduct, alongside Meta II, then gradually releases the retinal chromophore from the binding pocket leading to the appearance of free opsin. We monitored the kinetics of Meta III formation and decay by measuring the absorbance changes at 465 nm by means of UV-vis spectroscopy. First, the samples were left untreated and data were recorded for 10 min. After 10 min, the samples were photobleached for 30 s using the same light source indicated above, and absorbance data were continuously recorded for another 110 min, with one data point collected every 2 min. The results were analyzed by fitting the absorbance change at 465 nm to exponential functions. The t_1/2_ parameters for both the formation and decay of Meta III were determined from the fitted curves.

### Molecular structures of rhodopsin

The structure of bovine rhodopsin was visualized by means of PyMol using the 1L9H.pdb crystal structure from the Protein Data Bank. The molecular model was used as a backbone to highlight the amino acids that are different between mouse and human in all transmembrane regions of the receptor. The environment surrounding F88 was also visualized.

### Light microscopy

Light microscopy procedures have been described earlier^21^. Four-month-old mice were sacrificed by CO_2_ asphyxiation, and their eyes were enucleated and immersion-fixed for 24 h in phosphate-buffered saline (PBS, pH 7.4) containing 2% glutaraldehyde and 2% paraformaldehyde, at 4 °C. After dehydration, eyecups were embedded in an EPON-Araldite mixture and 5-μm sections were cut dorsal to ventral through the optic nerve and stained with hematoxylin and eosin. Images were acquired from the central retina (~500 μm from the optic nerve head) with a BZ-X800 (Keyence) microscope.

### *In vivo* retinal imaging

Following pupil dilation with 1% tropicamide and 2.5% phenylephrine (Akorn), 15-month-old mice were anesthetized with an IP injection of ketamine/xylazine solution (100/10 mg/kg). Ultrahigh-resolution spectral domain optical coherence tomography (OCT) (Bioptigen, Leica Microsystems) was performed for cross-sectional imaging of mouse retinas, as described previously^22^. Briefly, five frames of OCT images from each eye were first acquired in the B-mode in two orthogonal directions and then averaged. Images were analyzed for outer nuclear layer (ONL) thickness at 500 μm from the optic nerve head in four retinal quadrants (superior, inferior, nasal, temporal) using ImageJ 1.54p software and averaged to give an overall value per eye. The values from the right and left eye of the same animal were further averaged.

### Microspectrophotometry (MSP) from mouse retinas

The custom designed, Cornwall microspectrophotometer was used to measure the absorbance of mouse rod outer segments^23,24^. Retinas were isolated under infrared illumination from mice that were dark-adapted overnight. A segment of retina was oriented photoreceptor side up on a plexiglass recording chamber with a glass cover-slip bottom, gently flattened and held in place with a slice anchor (Warner Instruments). The chamber was perfused continuously with Ames medium (MyBiosource) buffered with sodium bicarbonate that was equilibrated with a gas mixture of 95% O_2_ / 5% CO_2_ (pH 7.4) and heated to maintain the tissue at 34–37 °C.

Measurements were made by passing a wide probe beam through many (predominantly rod) outer segments at the edge of a piece of retina over the wavelength range of 370–750 nm with a 1 nm resolution. Absorbance was calculated as: $\mathrm{OD}={\text{log}}_{10}(I_{i}/I_{t}$), where OD is the optical density (absorbance), $I_{i}$ is the light transmitted through a cell free space adjacent to the outer segments, and $I_{t}$ is the light transmitted through the tissue. All absorption spectra (optical density vs. wavelength) were measured with the electric vector of the probe beam oriented parallel to the plane of the intracellular disks and perpendicular to the long axis of the outer segment. Generally, averages were taken for 10 pre-bleach sample scans, 5 post-bleach sample scans, and 10 baseline scans, respectively, to increase the signal-to-noise ratio of the data. After measuring the dark-adapted rhodopsin spectrum, spectra of the photointermediates were recorded as a function of time following a 1-min exposure to bright, 505 nm LED light that was calibrated with a silicon photodiode (UDT Instruments) to bleach >90% of the visual pigment. After recording post-bleach spectra for up to 71 min, the retina was re-exposed to the bright light at 505 nm, to bleach the remaining pigment. Baseline corrections were applied to each averaged spectrum by subtracting a line extrapolated from 650–750 nm for that spectrum. This approach was preferred over the subtraction of the second post-bleach spectrum which often introduced slow drifts in the baseline that occurred over the long duration of the experiment or additional short-wavelength spectral components.

The time courses of Meta II and III were monitored at their maximal absorbances (380 nm and 472 nm, respectively) and normalized to that of dark rod visual pigment (~500 nm) after adjusting for the 1.16-fold higher molar extinction coefficient of Meta II with respect to rhodopsin^25^. Variability of the post-bleach spectral data precluded the analyses of Meta II or Meta III time courses for each individual retina, so results were averaged across all retinas prior to fitting.

For Meta II, normalized absorbances at 380 nm were fitted with a double-exponential function:

(1) $$\mathrm{Meta}\;II=A\cdot \left(e^{\left(-\frac{t}{\tau 2}\right)}+e^{\left(-\frac{t}{\tau 1}\right)}\right),$$

where MetaII is the time course of metarhodopsin II, A is the peak amplitude, t is the time after the light exposure (min), and $\tau_{1}$ and $\tau_{2}$ are the time constants of a double-exponential Meta II decay (min).

For Meta III, normalized absorbances at 472 nm were fitted with a bi-exponential function:

(2) $$\mathrm{Meta}\;\mathrm{III}=A\cdot \left(e^{\left(-\frac{t}{\tau 2}\right)}-e^{\left(-\frac{t}{\tau 1}\right)}\right),$$

where MetaIII is the time course of metarhodopsin III, A is the peak amplitude, t is the time after the light exposure (min), and $\tau_{1}$ and $\tau_{2}$ are the time constants of Meta III formation and decay, respectively (min).

### Single-cell suction electrode recordings from mouse rods

Single-cell recordings were carried out as previously described^21^. Briefly, WT and F88L mutant animals were dark-adapted overnight and sacrificed by CO_2_ asphyxiation. Their retinas were then removed under infrared illumination, chopped into small pieces, and transferred into a perfusion chamber located on the stage of an inverted microscope. A single rod outer segment was drawn into a glass microelectrode filled with Locke’s solution containing 140 mM NaCl, 3.6 mM KCl, 2.4 mM MgCl_2_, 1.2 mM CaCl_2_, 3 mM HEPES (pH 7.4), 0.02 mM EDTA, and 10 mM glucose. The perfusion solution contained 112.5 mM NaCl, 3.6 mM KCl, 2.4 mM MgCl_2_, 1.2 mM CaCl_2_, 10 mM HEPES (pH 7.4), 20 mM NaHCO_3_, 3 mM Na succinate, 0.5 mM Na glutamate, 0.02 mM EDTA, 10 mM glucose, 0.2% (v/v) MEM Amino Acids (50x) solution (Sigma-Aldrich), and 0.1% (v/v) MEM Vitamin Solution (100x) (Sigma-Aldrich). The solution was continuously bubbled with a 95% O_2_ / 5% CO_2_ mixture and heated to 37–38 °C.

Test flashes (2 or 20-ms) of calibrated 500-nm light were delivered by custom-made LED system. The stimulating light intensity was controlled by an LED driver and neutral density filters. Intensity-response relationships were fitted with Naka-Rushton hyperbolic functions, as follows:

$$R=\frac{R_{max}\cdot I^{n}}{I^{n}+I_{1/2}^{n}},$$

where R is the transient-peak amplitude of the response, $R_{max}$ is the maximal response amplitude, I is the flash intensity, n is the Hill coefficient, and $I_{1/2}$ is the half-saturating light intensity.

Photoresponses were amplified, low-pass filtered (30 Hz, 8-pole Bessel), and digitized (1 kHz). Rod dim flash sensitivity ($S_{f}$) was calculated from the linear region of the intensity-response curve as the ratio of the response amplitude to a given flash strength. Normalized rod dim flash fractional sensitivity ($S_{f(n)}$) was calculated by normalizing S_f_ by the amplitude of the saturated response. Half-saturating light intensity ($I_{1/2}$) was obtained from the intensity-response fit above as the test flash intensity required to produce a response with an amplitude equal to half of the amplitude of the corresponding saturated response. Integration time ($T_{\mathrm{integr}}$) was calculated as the integral of the dim flash response with the transient peak amplitude normalized to unity. The time constant for the dim flash response recovery ($\tau_{\mathrm{rec}}$) was derived from the single-exponential fit to the declining phase of the response. The dominant recovery time constant ($\tau_{D}$) was determined from supersaturating flashes^26^, by using a 10% criterion for photocurrent recovery from saturation. Data were analyzed with Clampfit 10.6 software.

### *In vivo* electroretinography (ERG)

*In vivo* ERGs were recorded as described before^27^. Briefly, mice were dark-adapted overnight and anesthetized by IP injection of ketamine (100 mg/kg) and xylazine (4 mg/kg). Pupils were dilated with a drop of 1% atropine sulfate. Mouse body temperature was maintained at 37 °C with a heating pad. ERG a-wave and b-wave responses were measured from both eyes by contact corneal electrodes held in place by a drop of Gonak solution (Akorn). Full-field ERG responses to increasing light intensities were recorded with a UTAS BigShot apparatus (LKC Technologies) using Ganzfeld-derived test flashes of calibrated green 530 nm LED light (within a range from 2.2×10^−5^ cd∙s m^−2^ to 23.5 cd∙s m^−2^) or white light generated by the Xenon Flash tube (from 80.7 cd∙s m^−2^ to 700 cd∙s m^−2^), as previously described^28^.

For rod dark adaptation experiments, rod ERG a-wave fractional flash sensitivity ($S_{f}$) in WT and F88L mutant animals was first determined in the dark from the linear part of the intensity-response curve as follows:

$$S_{f}=\frac{A}{A_{max\cdot I}},$$

where A is the amplitude of the rod a-wave dim flash response, $A_{max}$ is the maximum amplitude of the rod a-wave response for that eye (determined at 23.5 cd∙s m^−2^), and I is the flash strength. To monitor the post-bleach recovery of rod ERG $A_{max}$ and $S_{f}$, over 90% of rhodopsin was bleached with a 35-s exposure to 520 nm LED light focused at the surface of the cornea. The bleached fraction was estimated from the following equation:

$$F=1-e^{\left(-I\cdot P\cdot t\right)},$$

where F is the fraction of pigment bleached, t is the duration of the light exposure (s), I is the bleaching light intensity of 520 nm LED light (1.3 × 10^8^ photons μm^−2^ s^−1^), and P is the photosensitivity of mouse photoreceptors at the wavelength of peak absorbance (5.7 × 10^−9^ μm^2^ for mouse rods^29^. Mice were re-anesthetized once 30 min after the bleach with ~1/3 of the initial dose of ketamine. If needed, a small drop of PBS solution was gently applied to their eyes with a plastic syringe to protect them from drying and maintain electrode contacts. For each time point, the $A_{max}$ and $S_{f}$ were finally normalized to the corresponding dark-adapted (DA) values, ${A_{\text{max}}}^{DA}$ and ${S_{f}}^{DA}$. Data were analyzed with EM for Windows 9.4.0 (LKC Technologies) and Origin 2025 software.

### *Ex vivo* ERG recordings from mouse retinas

Transretinal ERG recordings were performed as described earlier^30^. Briefly, mice were dark-adapted overnight and sacrificed by CO_2_ asphyxiation. The whole retina was removed from each mouse eyecup under infrared illumination and stored in oxygenated aqueous L15 (13.6 mg/ml, pH 7.4) solution (Sigma-Aldrich) containing 0.1% BSA, at room temperature. The retina was mounted on filter paper with the photoreceptor side up and placed in a custom-made perfusion chamber between two electrodes connected to a differential amplifier. The tissue was perfused with Locke’s solution (same as for single cell recordings, see above). The solution was supplemented with 2 mM L-glutamate and 10 μM DL-2-amino-4-phosphonobutyric acid to block postsynaptic components of the photoresponse^31^, and with 20 μM BaCl_2_ to suppress the slow glial PIII component^32^. The solution was continuously bubbled with a 95% O_2_/5% CO_2_ mixture and heated to 36–37 °C.

Photoreceptors in the retina were stimulated with 20-ms test flashes of calibrated 505 nm LED light. The light intensity was controlled by a computer and neutral density filters in 0.5 log unit steps. To monitor the post-bleach recovery of (predominantly) rod ERG a-wave flash sensitivity ($S_{f}$, see definition in a previous section), >90% of rhodopsin was bleached with a 3-s exposure to 505 nm light. The bleached fraction was estimated from the same equation as described for a similar dark adaptation experiment *in vivo* (see above). For the rest of the recordings, the retinas were kept in darkness and sensitivity was measured periodically with a test flash. Photoresponses were amplified by a differential amplifier (DP-311, Warner Instruments), low-pass filtered at 30 Hz (8-pole Bessel), and digitized at 1 kHz. Data were analyzed with Clampfit 10.6 and Origin 2025 software.

### Mouse optomotor responses

Visual acuity and contrast sensitivity of WT and F88L mutant mice were evaluated with the 4-computer monitor OptoDrum system designed for automated measurements (Striatech, Tübingen, Germany). Optomotor responses were determined under scotopic (4×10^−3^ cd m^−2^) or photopic (250 cd m^−2^) background illumination conditions. Scotopic conditions were achieved by placing neutral density film filters over the monitors. Visual acuity was defined as the threshold for spatial frequency ($F_{s}$) of sine-wave grating stimuli with 100% contrast and measured at a speed ($S_{p}$) of 6 deg/s (scotopic) or 12 deg/s (photopic). In the visual acuity measurements, $F_{s}$ was gradually increased by the computer protocol until its threshold was determined. Temporal frequency ($F_{t}$) was automatically adjusted by the computer program, based on the following equation: $F_{t}=S_{p}\cdot F_{s}$^33^. Contrast sensitivity was defined as the inverse of contrast threshold for optomotor responses. In this mode, contrast of the stimuli was gradually decreased by the computer protocol until reaching its threshold. $F_{s}$ was fixed at 0.128 cyc/deg (46 cycles), and $S_{p}$ was set to 6 deg/s (scotopic) or 12 deg/s (photopic). $F_{t}$ was automatically adjusted and fixed at 0.8 Hz (scotopic) or 1.5 Hz (photopic).

### Statistical analysis

All experimental data were expressed as mean ± SEM and analyzed with the independent two-tailed Student’s *t*-test (with an accepted significance level of *P* < 0.05).

## Results

### Normal UV-Vis spectroscopic properties of purified F88L rhodopsin

**Fig. 2.**
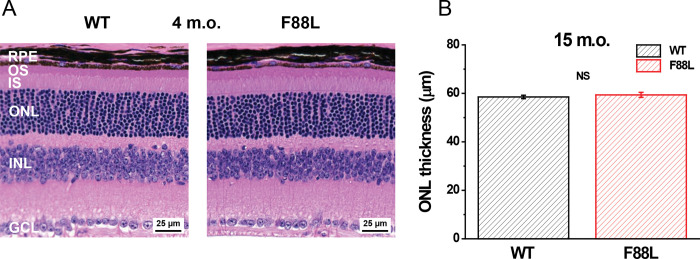
Retinal morphology in WT and F88L mutant mice. (**A**) Representative H&E-stained retinal cross-sections from 4-month-old animals. RPE: retinal pigment epithelium, OS: outer segments of photoreceptors, IS: inner segments of photoreceptors, ONL: outer nuclear layer, INL: inner nuclear layer, GCL: ganglion cell layer. Scale bars, 25 μm. (**B**) Normal ONL thickness in 15-month-old F88L mice measured by OCT. Values are means ± SEM (n = 5 for WT, n = 4 for F88L). NS: not significant (*P* > 0.05).

Using CRISPR/Cas9 technology, we created knock-in mutant mice with a F88L amino acid substitution in rhodopsin expressed under its native promoter that were then bred to homozygosity. Control animals were derived on the same genetic background. First, we analyzed whether the expression of F88L mutant visual pigment instead of rod’s native rhodopsin affected retinal structure. Examination of H&E-stained retinal sections revealed normal outer retina morphology in mutant mice. In 4-month-old animals, the thickness of the photoreceptor outer segment layer, the inner segment layer, the outer nuclear layer (ONL), as well as that of all inner retinal layers was comparable in control and F88L retinas (Fig. 2A). Even 15-month-old F88L mice exhibited normal ONL thickness, as measured by OCT (Fig. 2B). The unaltered retinal morphology in F88L mice allowed us to purify samples of similar rhodopsin content from dark-adapted mutant and WT mouse retinas which were dissected from the animals after they reached the age of 3–4 months.

**Fig. 3.**
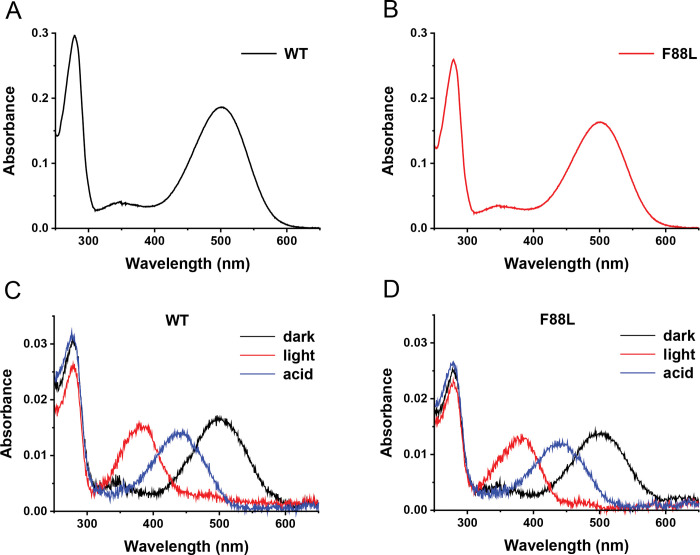
Spectroscopic analysis of WT and F88L mutant rhodopsins. Representative UV-visible spectra of immunopurified WT (**A**) and F88L (**B**) rhodopsins demonstrating the purity of samples. Photobleaching and acidification behavior of WT (**C**) and F88L mutant (**D**) rhodopsins. Representative dark spectra (black traces), photobleached spectra after 30-s illumination with green light (red traces) and spectra after immediate acidification of the sample following the photobleach (blue traces) are shown.

To begin a biochemical characterization of F88L mouse, the UV-Vis spectra of immunopurified WT and F88L rhodopsin obtained from two retinas were first measured in the dark state at 20 °C. The normal yield of the mutant rhodopsin obtained from F88L retinas (Fig. 3A, B) was consistent with their intact retinal structure. The recorded spectra were virtually identical for WT and F88L mutant, with the same spectral maximum of 500 nm and the same A_280_/A_500_ ratio of 1.6 (a clear indication of correctly folded rhodopsin). The lack of any spectral shift in the visible absorbance band for the F88L rhodopsin indicated the absence of any perturbation of the retinal binding pocket. This is consistent with the location of F88 position in rhodopsin pointing outside the helical bundle and away of the retinal binding site (Fig. 1B).

Notably, the WT and F88L rhodopsin mutant demonstrated an analogous photobleaching and acidification behavior. As noted above, in the dark state, the maximum absorption of the visible chromophoric band of both WT and F88L rhodopsin is located at 500 nm (black traces in Fig. 3C, D). After illumination, 11-*cis*-retinal photoisomerized to all-*trans*-retinal, resulting in a conformational change of the protein that adopted its active Meta II state, and a spectral shift of the visible band from 500 nm to 380 nm reflecting deprotonation of the retinal-opsin Schiff base linkage (red traces in Fig. 3C, D). Upon the immediate addition of sulfuric acid solution to the photoactivated protein, both rhodopsins denaturated and reprotonated the Schiff base nitrogen of Lys 296 corresponding to the covalent bond between all-*trans*-retinal and the unfolded polypeptide opsin chain, shifting the absorption band from 380 nm to 440 nm (blue traces in Fig. 3C, D). These results indicate that the first step of the phototransduction cascade was virtually unaffected by the F88L substitution.

### Increased thermal stability but normal chemical stability of F88L rhodopsin

**Fig. 4.**
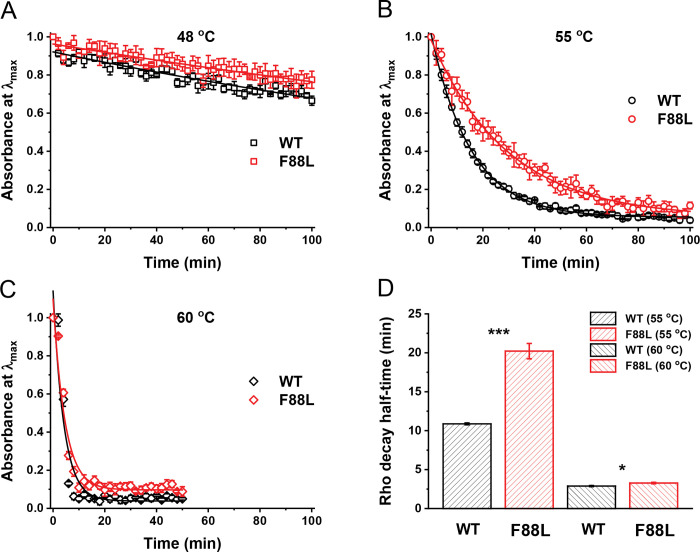
Thermal stability of WT and F88L mutant rhodopsins in the dark, measured by decrease in maximal absorbance (500 nm) at 48 °C (**A**), 55 °C (**B**), or 60 °C (**C**). Data were fitted with single exponential functions. (**D**) Rhodopsin half-life times derived from the fits in (**B–C**) (n = 3–4 for both pigments under each condition). *P < 0.05; ***P < 0.001.

The thermal stability of rhodopsin is an important indicator of its functional integrity. In some cases, amino acid substitutions in WT rhodopsin cause important stability changes like in mutations associated with the retinal degenerative disease retinitis pigmentosa (RP)^34^. Therefore, analyzing the stability of different rhodopsin variants provides relevant information on the conformational features of the proteins at different temperatures. We analyzed the thermal stability of WT and F88L rhodopsins at 48 °C, 55 °C, and 60 °C by UV-Vis spectroscopy and plotted the decrease of the 500 nm band over time (Fig. 4A–C). We found that at 48 °C, both F88L and WT rhodopsins did not exhibit significant thermal bleaching. After 100 min, ~80% of both rhodopsins remained intact. At 55 °C, only about 5% of the protein remained intact for both WT and F88L after 100 min; however, within this time frame, WT pigment denatured significantly faster than its F88L counterpart. Finally, at 60 °C, both WT and F88L rhodopsins were almost completely thermally bleached after approximately 20 min, and similar to the results at 55 °C, F88L pigment was more stable than WT. Based on the data in Fig. 4B, C, we calculated the t_1/2_ for the thermal bleaching process for WT and the F88L mutant (Fig. 4D). At 55 °C, the t_1/2_ values were 10.9 ± 0.1 min for WT and 20.2 ± 1.0 min for F88L (P = 0.0007), indicating significantly greater stability of the F88L pigment. At 60 °C, the t_1/2_ values were 2.9 ± 0.1 min for WT and 3.3 ± 0.1 min for F88L rhodopsin, respectively (P = 0.0351), demonstrating the extended range of increased thermal stability of the mutant.

In contrast to cone visual pigments, rhodopsin is very resistant to chemical bleaching by hydroxylamine. This small molecule agent can chemically bleach photoactivated forms of the rod pigment, but its access to 11-*cis*-retinal is sterically blocked in the ground state of rhodopsin. Using UV-Vis spectroscopy, we compared the stability of dark WT and F88L mutant pigments in presence of 50 mM hydroxylamine, at 20 °C and at 37 °C. We found that the final percentages of WT and mutant rhodopsin remaining after 60 min treatment were comparable at both temperatures (Table 1). This, together with its normal absorption spectrum, argues for the preservation of the compact structure of the chromophore-binding pocket in the dark F88L rhodopsin.

### Accelerated Meta II decay of F88L rhodopsin *in vitro*

**Fig. 5.**
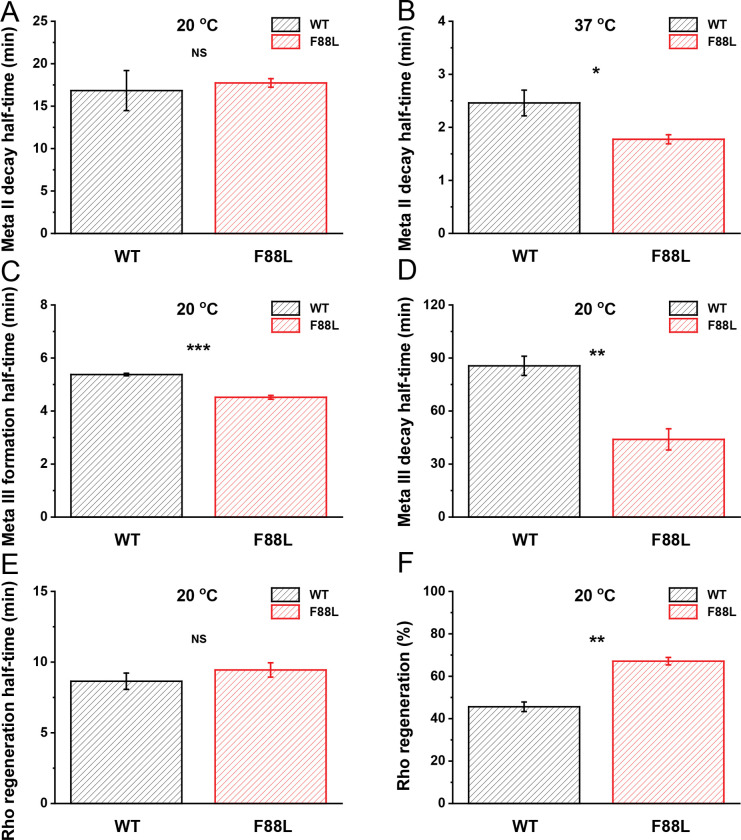
Decay of Meta II at 20 °C (**A**) or 37 °C (**B**), formation (**C**) and decay (**D**) of Meta III at 20 °C, and regeneration of rhodopsin with exogenous 11-*cis*-retinal at 20 °C (**E**) after near complete bleach for purified WT (n = 3–5) and F88L mutant (n = 3–4) pigments followed by fluorescence spectroscopy (for W265). Rhodopsin Meta II half-life times were derived from single exponential fits to the raw data. (**F**) Final levels of regenerated rhodopsin from experiments shown in (**E**). *P < 0.05; **P < 0.01; ***P < 0.001; *NS:* not significant (*P* > 0.05).

In the ground state of rhodopsin, 11-*cis*-retinal acts as an inverse agonist, stabilizing the inactive conformation of the protein. Upon light activation, the chromophore is converted to all-*trans*-retinal within femtoseconds and triggers conformational changes that result in the formation of the active Meta II state with a deprotonated Schiff base linkage. This is followed by Meta II decay, a process where all-*trans*-retinal leaves the chromophore-binding pocket of the opsin. This process releases the fluorescence by tryptophan at position 265 (W265) which can be measured to evaluate the rate of Meta II decay *in vitro*. Using this approach, we next monitored the chromophore release after photobleaching of the purified pigment at 20 °C or 37 °C and calculated the t_1/2_ of the Meta II decay from single-exponential fits to the data (Fig. 5A, B). We found that at 20 °C, the t_1/2_ values for WT and F88L Meta II showed no significant difference (16.8 ± 2.4 min and 17.7 ± 0.5 min, respectively, P > 0.05). However, at 37 °C, the t_1/2_ values were 2.6 ± 0.3 min and 1.8 ± 0.1 min for WT and F88L pigments, correspondingly (P = 0.032). Therefore, the active Meta II conformation of the F88L mutant decayed significantly faster at physiological temperature.

### Accelerated Meta III formation and decay of F88L pigment *in vitro*

Photolysis of WT and F88L pigments leads to the formation of the Meta II intermediate, which then partially transitions to Meta III before finally releasing all-*trans*-retinal and forming free opsin. In general, most studies have focused on the Meta II process, but the long-lasting Meta III byproduct is also crucial in the photocycle as it determines the overall production rate of unliganded opsin which becomes available for subsequent pigment regeneration. Therefore, we investigated the specific differences in the overall time course of Meta III between WT and F88L mutant at 20 °C (the data at 37 °C were unreliable in our conditions), by measuring absorbance changes for samples in solution, at 465 nm. In both WT and F88L samples, the absorbance at 465 nm first underwent a transient rise followed by a short plateau and finally declined. For the rising phase (reflecting Meta III production), the t_1/2_ values for WT and F88L pigments were 5.4 ± 0.05 min and 4.5 ± 0.07 min, respectively (Fig. 5C). For the decay phase, the t_1/2_ values for WT and F88L mutant were 85.6 ± 5.4 min and 44.0 ± 6.0 min, respectively, (Fig. 5D). Thus, both the formation and decay of Meta III were significantly faster in the F88L rhodopsin compared to those in WT under the *in vitro* conditions.

### More efficient regeneration of F88L rhodopsin *in vitro*

The regeneration of rhodopsin is crucial for maintaining scotopic (low-light) vision in vertebrate organisms. After rhodopsin photoactivation followed by chromophore release, the free opsin must recombine with a new 11-*cis*-retinal molecule to regenerate the ground pigment state, which enables the next round of continuous visual perception. By using UV-Vis spectroscopy, we next assessed the F88L rhodopsin regeneration ability *in vitro* (Fig. 5E, F). At 20 °C, we determined that the t_1/2_ regeneration values were not statistically different between the WT (8.6 ± 0.6 min) and F88L (9.4 ± 0.5 min, P > 0.05) pigment variants. Yet, the mutant pigment showed a better overall degree of regeneration with exogenous chromophore (Fig. 5F). We limited the data collection to this temperature, as all our attempts to measure the pigment regeneration at 37 °C failed, even for WT pigment, presumably due to irreversible destabilization of the opsin in the detergent solution under these conditions.

### Accelerated decay of Meta II but not Meta III in F88L rods *ex vivo*

While informative, the analysis of time courses of metarhodopsins II and III performed in mild detergent extracts of purified visual pigments can provide only a limited (and, in certain cases, controversial) information about their extrapolated rates of production and decay under physiological conditions. The limitations of *in vitro* approaches stem from documented effects of detergents on individual reactions of rhodopsin bleaching^35^ and regeneration^36^, as well as from the reasonable assumption that the parameters of various steps of the visual cycle and their possible regulatory mechanisms can strongly depend on lipid microenvironment in the membrane and morphological integrity of the cell. For instance, in our experiments, the normal decay of Meta II state of F88L pigment at 20 °C (Fig. 5A) was inconsistent with the accelerated kinetics of Meta III production at the same temperature (Fig. 5C). Furthermore, the decay of Meta III could not be determined reliably in our spectral measurements at 37 °C, where the chromophore release from its precursor, Meta II, was found to be faster in the mutant samples (Fig. 5B). Therefore, we also compared, by MSP recordings, the formation and decay of these two important rhodopsin photointermediates in intact WT and F88L mutant rods located on the edges of perfused retinal tissue, at the mouse physiological temperature.

**Fig. 6.**
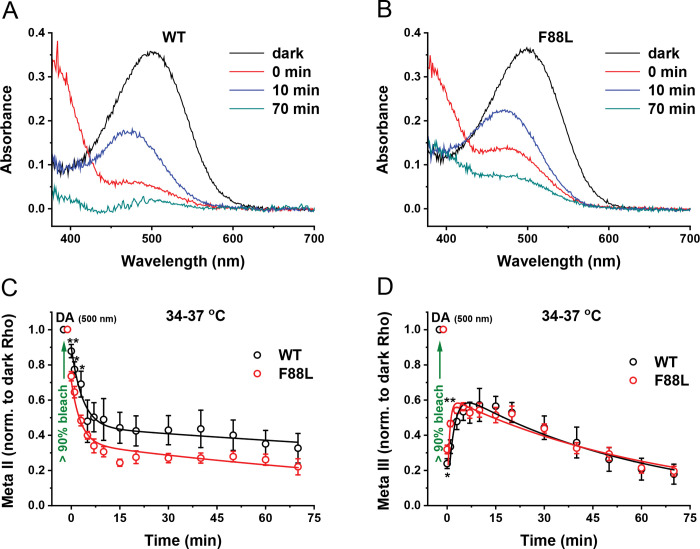
Microspectrophotometric characterization of metarhodopsins production and decay in WT and F88L mutant mouse retinas. Baseline-corrected absorbance spectra (transverse polarization of measuring beam light) of rhodopsin and its photointermediates Meta II and Meta III in WT (**A**) and F88L mutant (**B**) rods from typical MSP recordings. In (**A–B**), raw spectra were recorded from dark-adapted retinas (black), immediately after >90% bleach of the visual pigment with 505 nm light (red), 10 min after the bleach (blue) and 70 min after bleach (cyan). All recordings were made at 34–37 °C. (**C**) The time course of Meta II decay in WT (n = 5) and F88L (n = 7) retinas was determined at its maximal absorbance (380 nm) following >90% pigment bleach and normalized to that of dark rhodopsin (500 nm). The data were fitted with double-exponential functions, as indicated in Materials and Methods. (**D**) The time course of Meta III formation and decay in the same WT (n = 5) and F88L (n = 7) bleached retinas was measured at its maximal absorbance (472 nm) and normalized to that of dark rhodopsin (500 nm). The data were fitted with bi-exponential functions, as described in Materials and Methods. *P < 0.05; **P < 0.01; *NS:* not significant (*P* > 0.05, for all other points).

We obtained a series of absorption spectra of rhodopsin and its metaproducts in WT and F88L retinas before and after a 1-min exposure to green light to bleach >90% of the pigment (Fig. 6A, B). Similar to the situation *in vitro* (Fig. 3A, B), the spectra of dark-adapted rhodopsin (absorbance maximum at near 500 nm, black lines) and Meta II recorded immediately after the bleach (absorbance maximum at 380 nm, red traces) were comparable between WT and F88L lines. A 480-nm shoulder visible in the Meta II spectra mostly represents a mixture of Meta I (that is in fast equilibrium with Meta II) and photoregenerated (“unbleached”) fraction of rhodopsin^37^. In addition, given that our spectral scans required up to 30–40 s to complete, there is likely a contamination with a small amount of already formed Meta III. Spectra of the longest-lived Meta III intermediate (absorbance maximum at 472 nm, blue traces) recorded at the time of its peak, 10 min after the bleach, were also similar in WT and F88L rods. Meta II and Meta III progressively decayed to all-*trans*-retinal and opsin and largely disappeared after 70 min post-bleach (cyan traces, containing mostly “unbleached” fraction of rhodopsin).

The time course of Meta II decay was best described by a double-exponential function (see Materials and Methods). In agreement with our biochemical measurements (Fig. 5B), this process was accelerated in F88L mutant rods *ex vivo* (Fig. 6C). The decay time constants (*τ*_*1*_ and *τ*_*2*_) derived from the fit to averaged data from F88L retinas were 2.9 min and 141 min, respectively, whereas in WT retinas they were markedly slower (3.5 min and 317 min). On the other hand, the overall time course of Meta III in mouse rods could be best approximated by the subtraction of two exponential components (Fig. 6D). Consistent with the faster decay of its immediate precursor, Meta II (both *in vitro* and *ex vivo*), the production of Meta III in intact F88L rods was substantially faster than in WT counterparts (*τ*_*1*_ of 0.7 min vs. 1.9 min, correspondingly). However, we found that the mutant Meta III decayed with kinetics similar or slightly slower to that in WT under these *ex vivo* conditions (*τ*_*2*_ of 67 min vs. 58 min, respectively). This warrants that the interpretation of our results for the Meta III decay obtained at 20 °C *in vitro* (Fig. 5D) should be taken with caution.

Overall, the F88L mutant rhodopsin demonstrated normal spectral properties in its ground and post-bleach states, while possessed with elevated thermal stability, faster Meta II decay, and increased regenerability with 11-*cis*-retinal *in vitro*. The decay of Meta II was also accelerated *ex vivo*. To evaluate whether those altered pigment properties could affect the functional characteristics of rod photoreceptors, we next performed rigorous physiological and visual behavioral analyses of the F88L mutant mice.

### Normal retinal function and rod phototransduction in F88L mutant mice

**Fig. 7.**
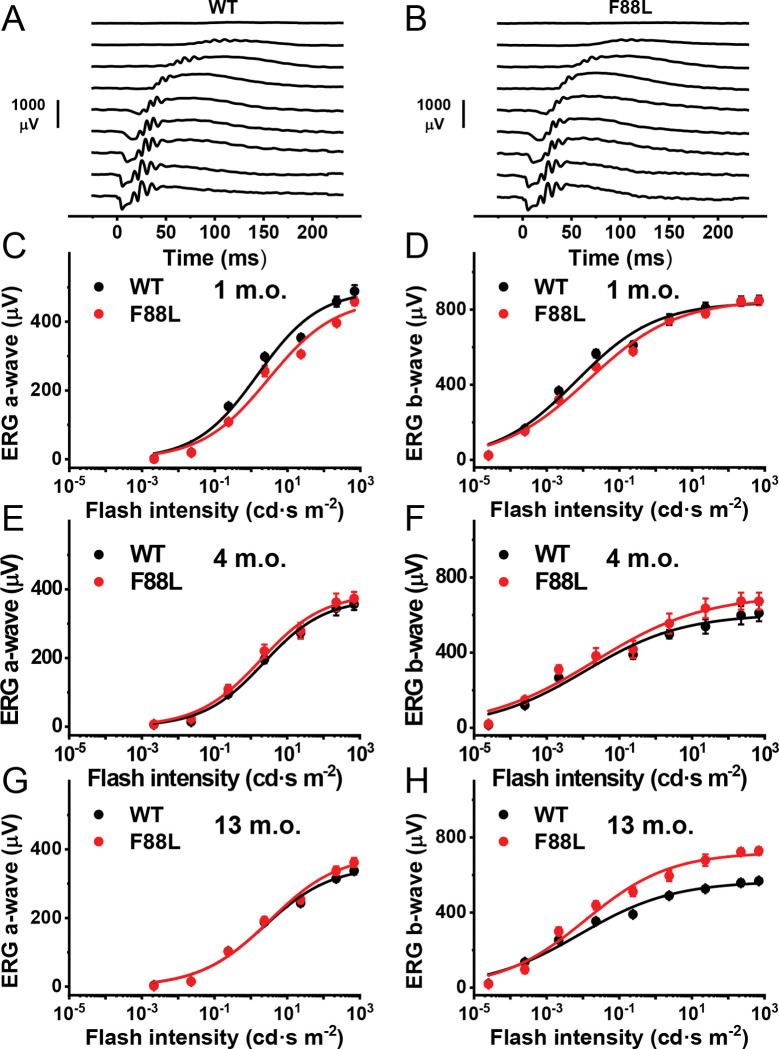
Rod visual function in WT and F88L mutant mice. Representative families of scotopic *in vivo* ERG responses of WT (**A**) and F88L mutant (**B**) mice. Test flash stimuli ranged from 2.2×10^−5^ cd∙s m^−2^ (top traces) to 700 cd∙s m^−2^ (bottom traces). Averaged scotopic ERG a-wave and b-wave intensity-response functions for dark-adapted WT mice (n = 3–4) and F88L mice (n = 3–4) at 1 month (**C–D**), 4 months (**E–F**), and 13 months (**G–H**) of age. Data in (**C–H**) were fitted with hyperbolic Naka-Rushton functions. Error bars for some points in (**C–H**) are smaller than the symbol size.

To begin physiological characterization of F88L mutant mice, we investigated whether the expression of F88L rhodopsin affects the ability of rods to produce light responses. *In vivo* ERG recordings indicated the normal scotopic (rod) visual function in F88L animals (Fig. 7A–H). Both rod-driven ERG a-wave and rod ON bipolar cell-driven ERG b-wave were essentially normal for up to 13 months of age in F88L mutant mice. Unexpectedly, in 13-month-old F88L animals the amplitude of the scotopic ERG b-wave was slightly but significantly larger than in the control mice (Fig. 7H). The reason for this change is unclear.

**Fig. 8.**
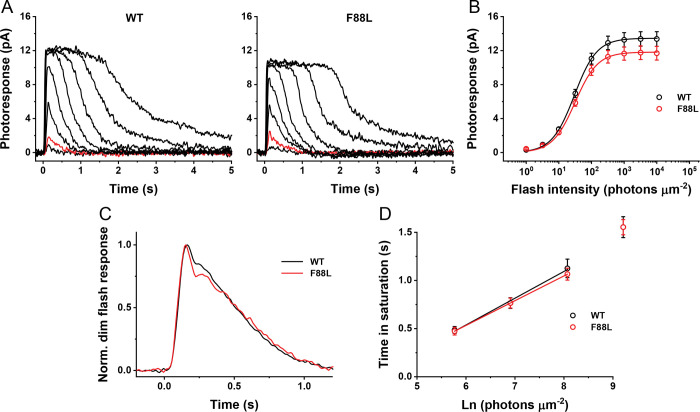
Photoresponses of 2-month-old WT and F88L mutant mouse rods. (**A**) Representative families of flash responses from WT (left) and F88L (right) mouse rods. Test flashes of 500 nm light with intensities of 3.2, 10, 32, 100, 320, 1000, 3200, and 10000 photons μm^−2^ were delivered at time 0. Red traces show responses to 10 photons μm^−2^ stimuli. (**B**) Averaged intensity-response functions for WT (n = 15) and F88L (n = 15) rods. Hyperbolic Naka-Rushton fits to the data yielded half-saturating intensities (*I*_*1/2*_) of 30 ± 1 and 31 ± 2 photons μm^−2^ for control and mutant rods, respectively (*NS*, *P* > 0.05). (**C**) Kinetics of phototransduction activation and inactivation in WT (n = 13) and F88L (n = 14) rods. Population-averaged, dim flash responses to light intensity of 10 photons μm^−2^ were normalized to their own peak amplitudes. (**D**) Determination of the dominant recovery time constant (τ_D_, n = 15 for both WT and F88L) from a series of supersaturating flashes. Linear fits to the data yielded τ_D_-values of 278 ± 31 ms and 257 ± 13 ms for control and mutant rods, respectively (*NS*, *P* > 0.05).

To determine whether the F88L rhodopsin mutation alters the activation and inactivation of the phototransduction cascade in mouse rods in more detail, we performed single-cell recordings from dark-adapted rods with a suction electrode (Fig. 8). In agreement with the similar lengths of their outer segments, dark-adapted control and F88L rods generated responses of comparable amplitudes (Fig. 8A, B, and Table 2). The dim flash responses of mutant rods were also comparable to those of control cells. Both phototransduction activation, measured from the rising phase of the dim flash response, and its late inactivation, characterized by the response recovery time constant (*τ*_*rec*_) were unaffected by the F88L mutation (Fig. 8C and Table 2). Finally, response recovery following a series of supersaturating flashes was also normal in F88L rods, as indicated by the comparable dominant recovery time constants (*τ*_*D*_) (Fig. 8D and Table 2).

Taken together, these findings confirm that the expression of rhodopsin F88L mutant in mouse rods neither compromises their signaling under dark-adapted conditions nor affects the overall rod health.

### Normal dark adaptation of rods in F88L mutant mice

The accelerated decay of mutant Meta II rhodopsin at 37 °C (Fig. 6C) and its potentially better regeneration with the exogenous 11-*cis*-retinal *in vitro* (Fig. 5E, F) motivated us to further investigate whether the F88L mutation could speed up the dark adaptation of mouse rods under physiological conditions. Therefore, we next determined the kinetics of rod dark adaptation by ERG *in vivo* by tracking the recovery of rod ERG a-wave amplitude and dim flash sensitivity after nearly complete (>90%) bleaching of the rod visual pigment. As determined by multiple prior studies, the dark adaptation of rods in the intact vertebrate eye is driven by the decay of photoactivated visual pigment and its subsequent regeneration with fresh 11-*cis*-chromophore supplied by the retinal pigment epithelium (RPE).

**Fig. 9.**
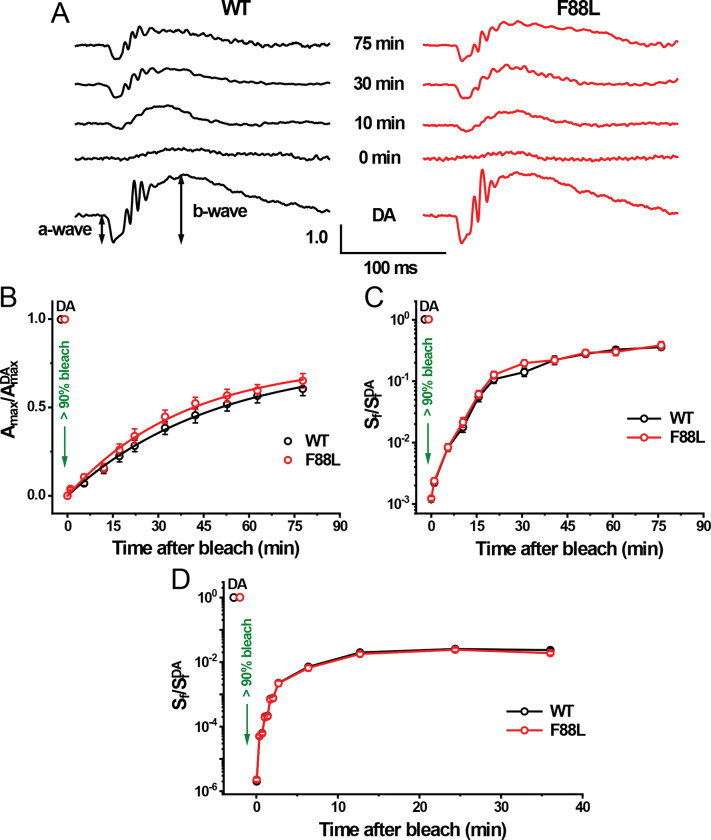
Dark adaptation of rods in WT and F88L mutant mice. (**A**) Representative scotopic *in vivo* ERG responses in the dark (dark-adapted [DA], bottom) and at indicated times after bleaching >90% of the rod pigment (520-nm LED light for 35 s) in 2-month-old WT (left) and F88L mutant (right) mice. For each time point, rod ERG a-wave amplitude ($A_{\text{max}}$) values were normalized to their corresponding dark-adapted maximal rod response ($A_{\text{max}}$^DA^). Recovery of normalized $A_{\text{max}}$ (**B**) and flash sensitivity (*S*_f_) (**C**) after bleaching >90% rhodopsin at time 0 in the same WT (n = 7) and F88L (n = 9) animals. Data points in (**B**) were fitted with single exponential functions that yielded time constants of 49 ± 4 min and 39 ± 4 min, respectively. ${S_{f}}^{\text{DA}}$ in (**C**) refers to pre-bleach sensitivity in the dark. Error bars for some points in (**C**) are smaller than the symbol size. (**D**) *Ex vivo* recovery of normalized (predominantly rod) ERG a-wave flash sensitivity ($S_{f}$) in isolated retinas of WT (n = 8) and F88L (n = 8) animals after bleaching >90% of rhodopsin at time 0 with 505 nm LED light. $S_{f}$^DA^ denotes the sensitivity of dark-adapted (DA) rods. Results for WT and F88L in (**B–D**) were not significantly different, *P* > 0.05, for all points.

First, we recorded rod-driven dark ERG responses and found that they were of similar waveforms and maximal a-wave amplitudes in WT (304 ± 15 μV, n = 7 mice) and F88L mutant mice (292 ± 8 μV, n = 9 mice, P > 0.05) (Fig. 9A). Animals’ eyes were then exposed to bright green light to bleach the bulk of their pigment, and the recovery of their flash responses was monitored in the dark. Immediately after a nearly complete pigment bleach, rods in both control and mutant mice produced barely detectable a-wave responses that were desensitized by ~3 log units. Over the following 75 min of dark adaptation, photoresponses in both lines recovered gradually (Fig. 9A). The recovery of the averaged maximal ERG a-wave amplitude to a saturating flash intensity ($A_{\text{max}}$) in WT rods could be described by a single exponential function with a time constant of 49 ± 4 min, and its final level 75 min after the bleach was ~61% of its pre-bleach dark-adapted value (Fig. 9B, black symbols). Although the rod dark adaptation of F88L mice appeared to be slightly faster and more complete (~65% response recovery by 75 min), its rate (39 ± 4 min) was not significantly different from that of control rods (Fig. 9B, red symbols, P > 0.05 at any post-bleach time point). Similarly, no acceleration was observed in the recovery of mutant rod-driven ERG a-wave sensitivity (S_f_) following the same bleach (Fig. 9C). These results demonstrate that the F88L substitution alone was not sufficient to speed up detectably the recovery of rod function, driven by rhodopsin regeneration, after exposure to bright light *in vivo*. The outcome of our physiological experiment may not be surprising though, given that in normal, non-pathological, conditions the rate-limiting step in rod chromophore recycling lies outside photoreceptors, in the interphotoreceptor matrix and/or the RPE^38^.

Moreover, rods expressing the F88L rhodopsin recovered their sensitivity with normal kinetics even in isolated perfused retinas in which the synaptic transmission was blocked (Fig. 9D). Under these *ex vivo* conditions, the overall recovery process was at least ~50 times less efficient compared to that typically observed in the intact mouse eye. In this setting, with the lack of RPE-driven 11-*cis*-retinal recycling, the recovery is believed to be dominated by the gradual decay of rhodopsin intermediates (Meta II and Meta III) to all-*trans*-retinal and unliganded opsin^39^. The final post-bleach sensitivity level of rod photoreceptors under these conditions is determined by the steady-state level of free opsin^40,41^. Thus, the faster Meta II decay of F88L mutant pigment alone did not improve the bleaching adaptation of mouse rods *ex vivo*, when the most long-lived photoproduct (Meta III) was still decaying normally (Fig. 8D).

### Normal vision in F88L mutant mice

Finally, to explore the possibility that the F88L rhodopsin mutation could still have an effect on the overall visual performance of aged mice, we compared the visual acuity and contrast sensitivity in 15-month-old WT and mutant animals, by measuring optomotor head-turning responses to rotating grating stimuli^33,42^. We found that the F88L mice had normal visual acuity and contrast sensitivity under dim light conditions in which vision is dominated by rods (Table 3). Similarly, under bright light conditions in which vision is dominated by cones, the mutant mice performed just as well as WT controls (Table 3). These results are in agreement with the well-preserved retinal morphology in F88L mutants even at 15 months of age (Fig. 2B). Interestingly, these findings also indicate that the observed increase in the ERG b-wave (Fig. 7H) in these old animals does not improve their overall vision.

## Discussion

The protein amino acid sequence shifts among closely related species reflects adaptation to their different environmental conditions. In the case of rhodopsin, adaptation to light conditions (diurnal versus nocturnal) is an important evolutionary factor to be considered in this kind of analysis. Here, we have conducted a detailed molecular characterization of “humanized” Leu-88 rhodopsin variant obtained from a knock-in mutant mice and performed a rigorous analysis of the visual function of animals carrying this mutation by morphological, electrophysiological, and behavioral methods.

Fig. 1 illustrates the positions of amino acids different between mouse and human rhodopsin superimposed on the solved bovine rhodopsin three-dimensional structure. The differences in amino acid composition between these two species is limited to only 18 residues out of the total 348 amino acids in the full-length rhodopsin sequence (Fig. 1A). Furthermore, most of these changes preserve their physicochemical properties. The position 88 in the 2^nd^ rhodopsin transmembrane domain is occupied by phenylalanine (Phe) in the mouse pigment, whereas it is a leucine (Leu) in the human pigment (Fig. 1B). These two amino acids have a marked hydrophobic character and although Phe is relatively bulkier, the difference in their sizes is not dramatic. Therefore, one would expect that the F88L mutation alone should have little effect on the protein conformational properties. However, our molecular analysis conducted on the protein purified from mouse retinas showed that the F88L mutant is more thermally stable than the WT mouse rhodopsin, suggesting that this single amino acid switch contributes to the higher stability of the human *vs.* mouse pigments^43^.

The amino acid 88 may also have a structural role in rhodopsin because the human Leu-88 to Pro mutation leads to a severe early-onset rod-cone dystrophy phenotype in patients^44^. Disease-causing mutations have also been reported for the surrounding residues, Val-87 to Asp, Gly-89 to Asp or to Lys, Gly-90 to Asp or to Val, and opsin misfolding or loss of stability has been hypothesized as their pathogenic mechanism^20,45–50^. Leu-88 is located within the α-helix of the 2^nd^ transmembrane domain of rhodopsin. The residue at this position is not invariant among Metazoan organisms but always shows hydrophobic characteristics, necessary for the maintenance of this α-helix. The substitution of the Leu by a Pro would induce a kink in the helix and destabilize the protein through opsin misfolding. This would classify the Leu-88 to Pro mutation within a disease class II, after Mendes and colleagues^51^. In our case, the physicochemical nature of the F88L change is rather conservative, corresponding to two hydrophobic residues with comparable size, so no significant charge or steric effects are expected from the substitution. The observed increased thermal stability could be the result of improved packing of Leu in the hydrophobic environment of helix II where it is located or altered interaction with the lipid bilayer in the disc membrane. In this regard, it has been proposed that Leu has a better propensity to be accommodated in an α-helix than Phe^52^.

The rate of rhodopsin resetting following its activation by light is thought to be an adaptive feature of the visual system when it has to react quickly to rapid changes in illumination of the environment that species inhabit^53^. It has been proposed that the earliest vertebrates living in shallow waters were likely diurnal, and that the gradual transition to nocturnality occurred later in their evolution^54^. This evolutional switch to scotopic vision has been recently investigated by the analysis of reconstructed rod visual pigments from ancestral taxa. It has been suggested that, in addition to relatively minor adjustments in rhodopsin’s spectral properties, it was accompanied by adaptive slowing in the rate of retinal release from its photoactivated state, Meta II^55^. with the most dramatic shifts occurring when the early vertebrates began occupying a terrestrial niche and became Tetrapoda and later, Mammalia and Theria^56^. While the exact role of position 88 in rhodopsin evolution within/post-Theria remains to be determined, exciting recent evidence suggests its involvement in the secondary transition to crepuscularity (twilight visual niche) by at least two independent animal groups: ancestral monotremes (extant representatives – echidnas and platypus) and an extinct branch of crocodilians^57^. Guo and co-authors have found that in both groups, the F88L mutation resulted in substantial acceleration of retinal release rate from Meta II *in vitro*^57^. For monotremes, two other mutations (V81F and L84H) causing similar accelerating effect and located in the same hydrophobic rhodopsin helix (Fig. 1B) were identified in that study, indicating the importance of this region in regulating the lifetime of photoactivated rhodopsin. Interestingly, these two amino acid positions are conserved between mouse and human (and bovine) rhodopsins, thus leaving the residue at position 88 as the only determinant (in this part of opsin) of faster Meta II decay of F88L mutant pigment compared to its WT mouse counterpart, found in our study (Fig. 5B and 6C). Whether in cone opsins the homologous residue(s) of this position (Val or Ile in human and mouse) play a role in the dramatically faster retinal release^58–60^ alongside the already established cone residues at positions 189 and 122^61^ would be an interesting subject of future research.

Notably, despite the accelerated chromophore release and higher thermal stability of “humanized” mouse F88L pigment, the basic visual function of mice carrying the F88L substitution appeared to be quite normal, even in animals near the end of their lifespan (Fig. 7G–H and Table 3). Moreover, the differences in the rates of F88L Meta II decay observed both *in vitro* and *ex vivo*, as well as in the recombination with 11-*cis*-retinal found in our biochemical experiments were not sufficient to accelerate the kinetics of rod dark adaptation driven by pigment regeneration in living animals under physiological conditions, or even in isolated perfused retinas in which the decay rate of the most long-lived rhodopsin photoproduct (Meta III) was unaltered (Fig. 6D). Thus, a well-documented fact of more rapid rod dark adaptation in humans compared to mice and other nocturnal species^38,62^ remains to be explained by the synergistic effect of other differences in rhodopsin amino acid sequence, differences in the RPE visual cycle activity, or a combination of the two.

Our present results support the view of the rhodopsin structure as a sophisticated network of amino acid interactions and reinforces the notion that subtle substitutions in the protein sequence can translate into significant changes in the conformational stability of this key visual photoreceptor protein. This study also suggests that certain amino acid positions have played a critical role in the molecular evolution of rhodopsin and the environmental light adaptation of different mammalian species, ranging from mouse to human. In this specific case, our results argue that the position 88 in rhodopsin may have been involved in the molecular evolution of vision from nocturnal (mouse) back to diurnal (human) conditions. In this regard, the increased protein stability caused by the F88L mutation, together with the shortened lifetime of Meta II state and enhanced recombination with chromophore, point to a definite role of this residue in the molecular evolution of rhodopsin.

## Figures and Tables

**Table 1.** Chemical stability of rhodopsin under two different temperature conditions. Chemical stability of WT and F88L mutant rhodopsin under two different temperature conditions (20 °C or 37 °C). Rhodopsin was treated with hydroxylamine at a final concentration of 50 mM for 60 min. Values give the final percentages of the remaining pigment, determined from single exponential fits to the absorbance decreases at 500 nm.

20 °C		37 °C	
WT (n = 4)	F88L (n = 4)	WT (n = 4)	F88L (n = 4)
69.4 ± 3.6	67.8 ± 1.3	75.9 ± 1.9	81.0 ± 1.5
*P* > 0.05 (*NS*)		*P* > 0.05 (*NS*)	

**Table 2.** Parameters of rod single-cell responses. Parameters of rod single-cell responses from WT and F88L mice. I_dark_, dark current measured from saturated responses; time-to-peak (T_peak_) and integration time (T_integr._) refer to responses whose amplitudes were < 0.2-fold of I_dark_ and fell within the linear range; I_1/2_, half-saturating flash intensity; S_f,_ dim flash sensitivity (amplitude of dim flash response divided by flash strength); S_f(n),_ normalized S_f_ (calculated as above and then normalized to I_dark_); τ_rec_, time constant of single-exponential decay of dim flash response recovery phase; τ_D_, dominant time constant of recovery (the “Pepperberg constant”) determined as the slope of the linear fit of saturation time of bright flash responses vs. natural logarithm of flash intensity. (*NS*, not significant for all parameters).

Parameter	WT (n = 13–15)	F88L (n = 14–15)	*P*-value
I_dark_ (pA)	13 ± 1	12 ± 1	> 0.05 (*NS*)
T_peak_ (ms)	173 ± 12	189 ± 33	> 0.05 (*NS*)
T_integr._ (ms)	432 ± 38	434 ± 44	> 0.05 (*NS*)
I_1/2_ (ph/μm^2^)	30 ± 1	31 ± 2	> 0.05 (*NS*)
S_f_ (pA*μm^2^/ph)	0.28 ± 0.02	0.25 ± 0.03	> 0.05 (*NS*)
S_f(n)_ (μm^2^/ph)	0.021 ± 0.001	0.022 ± 0.003	> 0.05 (*NS*)
τ_rec_ (ms)	314 ± 36	357 ± 42	> 0.05 (*NS*)
τ_D_ (ms)	278 ± 31	257 ± 13	> 0.05 (*NS*)

**Table 3.** Parameters of mouse vision derived from optomotor responses. Parameters of mouse vision derived from optomotor responses. Mouse optomotor responses were measured under scotopic (4 × 10^−3^ cd m^−2^) or photopic (250 cd m^−2^) background illumination conditions in mice at 15 months of age (n = 5 for both WT and F88L). *NS:* not significant (*P* > 0.05).

Parameter	WT (n = 5)	F88L (n = 5)	*P*-value
Scotopic visual acuity (cyc/deg)	0.32 ± 0.02	0.32 ± 0.01	> 0.05 (*NS*)
Scotopic contrast sensitivity	15.11 ± 1.54	12.28 ± 1.03	> 0.05 (*NS*)
Photopic visual acuity (cyc/deg)	0.50 ± 0.08	0.49 ± 0.03	> 0.05 (*NS*)
Photopic contrast sensitivity	42.82 ± 3.91	46.74 ± 4.79	> 0.05 (*NS*)

## Data Availability

All data generated or analyzed during this study are included in this article. Additional data may be available from corresponding authors upon reasonable request.
